# Identification of a set of miRNAs differentially expressed in transiently TIA-depleted HeLa cells by genome-wide profiling

**DOI:** 10.1186/1471-2199-14-4

**Published:** 2013-02-06

**Authors:** Carmen Sánchez-Jiménez, Isabel Carrascoso, Juan Barrero, José M Izquierdo

**Affiliations:** 1 Centro de Biología Molecular Severo Ochoa, Consejo Superior de Investigaciones Científicas, Universidad Autónoma de Madrid (CSIC/UAM), C/Nicolás Cabrera 1, Cantoblanco, Madrid 28049, Spain; 2 Current address: Programa de Biología de Sistemas, Centro Nacional de Biotecnología, Consejo Superior de Investigaciones Científicas, C/Darwin 3, Cantoblanco, Madrid, 28049, Spain

**Keywords:** TIA1, TIAR, miRNAs, Gene regulatory networks

## Abstract

**Background:**

T-cell intracellular antigen (TIA) proteins function as regulators of cell homeostasis. These proteins control gene expression globally at multiple levels in response to dynamic regulatory changes and environmental stresses. Herein we identified a micro(mi)RNA signature associated to transiently TIA-depleted HeLa cells and analyzed the potential role of miRNAs combining genome-wide analysis data on mRNA and miRNA profiles.

**Results:**

Using high-throughput miRNA expression profiling, transient depletion of TIA-proteins in HeLa cells was observed to promote significant and reproducible changes affecting to a pool of up-regulated miRNAs involving miR-30b-3p, miR125a-3p, miR-193a-5p, miR-197-3p, miR-203a, miR-210, miR-371-5p, miR-373-5p, miR-483-5p, miR-492, miR-498, miR-503-5p, miR-572, miR-586, miR-612, miR-615-3p, miR-623, miR-625-5p, miR-629-5p, miR-638, miR-658, miR-663a, miR-671-5p, miR-769-3p and miR-744-5p. Some up-regulated and unchanged miRNAs were validated and previous results confirmed by reverse transcription and real time PCR. By target prediction of the miRNAs and combined analysis of the genome-wide expression profiles identified in TIA-depleted HeLa cells, we detected connections between up-regulated miRNAs and potential target genes. Gene Ontology (GO) and Kyoto Encyclopedia of Genes and Genomes (KEGG) database analysis suggest that target genes are related with biological processes associated to the regulation of DNA-dependent transcription, signal transduction and multicellular organismal development as well as with the enrichment of pathways involved in cancer, focal adhesion, regulation of actin cytoskeleton, endocytosis and MAPK and Wnt signaling pathways, respectively. When the collection of experimentally defined differentially expressed genes in TIA-depleted HeLa cells was intersected with potential target genes only 7 out of 68 (10%) up- and 71 out of 328 (22%) down-regulated genes were shared. GO and KEGG database analyses showed that the enrichment categories of biological processes and cellular pathways were related with innate immune response, signal transduction, response to interleukin-1, glomerular basement membrane development as well as neuroactive ligand-receptor interaction, endocytosis, lysosomes and apoptosis, respectively.

**Conclusion:**

All this considered, these observations suggest that individual miRNAs could act as potential mediators of the epigenetic switch linking transcriptomic dynamics and cell phenotypes mediated by TIA proteins.

## Background

Nowadays, the central dogma of Molecular Biology —developed from classic research works aimed at determining the biology of prokaryotic organisms— is known to reflect only a part of the agenda containing the genetic information that gives rise to the complexity of eukaryotic organisms. The characterization of post-transcriptional events leading to the generation of multiple RNAs, proteins and functions from only one RNA precursor shows up the existence of multiple overlapping regulatory networks and mechanisms for the control of biological functions beyond transcriptional regulation. There is increasing evidence to support the idea that transcriptome and proteome regulation and heterogeneity are key stages to understand differences in the protein diversity observed in organisms of similar genetic complexity. It’s therefore necessary to fully characterize the modulators linking and synchronizing multiple layers of gene expression regulation.

T-cell intracellular antigen 1 (TIA1) and TIA1 related/like (TIAR/TIAL1) proteins are two DNA/RNA binding proteins that regulate many aspects of RNA metabolism at different levels. These multifunctional regulators can modulate: i) DNA-dependent transcription through its interaction with DNA and RNA polymerase II [1-4]; ii) alternative splicing of pre-mRNA through the selection of atypical 5^′^ spliced sites [5-8], and iii) stability and/or translation of eukaryotic mRNAs through the interaction with 5^′^ and/or 3^′^ untranslatable regions [9-16]. Some of these regulatory layers operate in the control of main biological programmes so as to maintain cellular homeostasis; this programmes include apoptosis, inflammation, cell responses to stress or viral infections ([10] and references included). Furthermore, mice lacking either TIA1 [16] or TIAR [17], as well as ectopically over-expressing TIAR [18], show higher rates of embryonic lethality.

MicroRNAs (miRNAs) are a class of 19-25 nt long non-coding RNAs that regulate post-transcriptionally gene expression by binding with partially complementary sequences on target mRNAs and inhibiting translation or affecting stability of these mRNAs [19]. Multiple lines of evidence indicate that they are key regulators of numerous critical functions in developmental, cell differentiation and disease processes, including tumorigenesis and cancer progression [19]. However, defining the place and function of miRNAs in complex regulatory networks is not straightforward. Systems’ approaches such as the inference of a module network from expression data can help to achieve this goal [19].

We have previously described specific changes of transcriptomic dynamics associated to inflammation, angiogenesis, metabolism, and cell proliferation-related genes upon TIA1/TIAR-RNA interference-based silencing in HeLa cells [10]. In the present study, we test the hypothesis whether there are specific changes associated to the pattern of miRNA expression which may interfere/modulate with target genes and, therefore, contribute to the phenotypes described in TIA-depleted HeLa cells. Herein, we identified a miRNA signature that is concomitant and coherent with biological processes and pathways associated to the phenotypes observed in HeLa cells lacking TIA proteins.

## Methods

### Cell culture and RNA interference (RNAi)analysis

HeLa cells were grown and transfected with 20 nM of either a control siRNA (non-silencing siRNA duplex fluorescein labeled 27-6411-02FL from Gene Link) or two siRNAs against TIA1 (5^′^-AAGCTCTAATTCTGCAACTCTTT-3^′^; 5^′^-AACAACTAA TGCGTCAGACTTTT-3^′^) and TIAR (5^′^-AAGTCCTTATACTTCAGTTGTTC-3^′^; 5^′^-AACCATGGAATCAACAAGGATTT-3^′^) directed to the positions 59-81/647-669 and 65-87/971-993 to the coding regions of TIA1 and TIAR mRNAs, respectively, as described previously [7,10].

### Cell extract preparation, western blot analysis and RNA purification

Whole-cell extracts were prepared by resuspensing the cells in lysis buffer (50 mM Tris–HCl, pH 8.0, 140 mM NaCl, 1.5 mM MgCl_2_, 0.5% Nonidet P-40 plus a mixture of protease inhibitors), freeze-thawing three times, and centrifugation at 10,000 rpm for 5 min. in a microfuge at 4°C. Resulting supernatants were recovered and stored at -70°C [10]. Immunoblots were carried out loading equal amounts of protein (15 μg) on 10% SDS-PAGE and using the following antibodies: anti-TIA1 and anti-TIAR from Santa Cruz Biotechnology (CA, USA) and anti-α-tubulin from Sigma (UK). RNA was extracted by using a miRVANA kit (Ambion, TX, USA) according to the manufacturer’s protocol.

### MicroRNA expression profiling analysis

The quality of the total RNA was verified by an Agilent 2100 Bioanalyzer profile. 2000 ng total RNA from each sample was labeled with Hy3™ or Hy5™ fluorescent label, using the miRCURY LNA™ microRNA Labeling Kit Hy3™/Hy5™ (Exiqon, Denmark), following the procedure described by the manufacturer. A Hy3™- and a Hy5™-labeled RNA sample were mixed pair-wise and hybridized to the miRCURY LNA™ microRNA Array (Exiqon, Denmark), which contains capture probes targeting all microRNAs for human registered in the miRBASE version 9.1. The hybridization was performed according to the miRCURY LNA™ microRNA Array Instruction manual using a Tecan HS4800™ hybridization station (Tecan, Austria). After hybridization the microarray slides were scanned and stored in an ozone free environment (ozone level below 2.0 ppb) in order to prevent potential bleaching of the fluorescent dyes. The miRCURY LNA™ microRNA Array slides were scanned using the Agilent G2565BA Microarray Scanner System (Agilent Technologies, Inc., USA) and the image analysis was carried out using the ImaGene® 7.0 (miRCURY LNA™ microRNA Array Analysis Software, Exiqon, Denmark). The quantified signals were background corrected (local background subtraction) and normalized using the global Lowess (LOcally WEighted Scatterplot Smoothing) regression algorithm.

Local background was corrected by normexp method with an offset of 50. Background corrected intensities were transformed to log scale (base 2) and normalized by Lowess for each array [20]. Finally, to have similar intensity distribution across all arrays, Lowess-normalized-intensity values were scaled by adjusting their quantiles [21]. After data processing each probe was tested for changes in expression over replicates using an empirical Bayes moderated t statistic [22]. To control the false discovery rate (FDR), P values were corrected using the method of Benjamini and Hochberg (1995) [23]. FIESTA viewer (http://bioinfogp.cnb.csic.es/tools/ FIESTA) was used to visualize all microarray results and to evaluate the numerical thresholds (-2> fold change >2; FDR < 0.0001) applied for selecting differentially expressed genes [24].

The miRPlus sequences are licensed human sequences (Exiqon, Denmark). Some of them are already annotated in the miRBase database version 18. Microarray data discussed in this publication have been deposited in the NCBI Gene Expression Omnibus database (http://www.ncbi.nlm.nih.gov/geo/info/linking.html) and are accessible through the GEO Series accession number GSE41213.

### QPCR

The method was optimized for microRNA, and reagents, primers, and probes were obtained from Applied Biosystems. Reverse transcriptase (RT) reactions and real-time PCR (PCR) were performed according to manufacturer protocols at the Genomic PCR Core Facility at Universidad Autónoma de Madrid in Madrid Scientific Park. Analyses were performed in two independent samples by triplicate, including no-template and RT-minus controls. U6 RNA and miR-200 expression were used as endogenous reference controls. Relative miRNA expression was calculated using the comparative cycle threshold method.

### Generation of miRNA targets dataset

In silico targets predicted for each of the differentially expressed miRNAs by three different algorithms: TargetScan 5.2 ([25] and references included), PicTar-Vert ([26] and references included) and miRDB [27,28] were downloaded using web-app miRBase [29] (http://www.mirbase.org). Given that each algorithm focus on different aspects of miRNA-mRNA pairing, and the lack of experimental validation of most miRNAs targets do not allow a false-positive elimination, we kept the datasets by considering the following score values: TargetScan 5.2 (aggregate PCT > 0.1), PicTar-Vert (PicTar score > 2) and miRBD (target score > 70).

### Gene ontology, pathway and network analyses

The Gene Ontology (GO) and Kyoto Encyclopedia of Genes and Genomes (KEGG) database analysis were conducted using software programmes provided by GenCodis3 (http://genecodis.cnb.csic.es) [30,31]. Networks and regulatory topologies of functional relationships between gene clusters were created using the CytoScape [32] (http://www.cytoscape.org).

## Results

**Figure 1:**
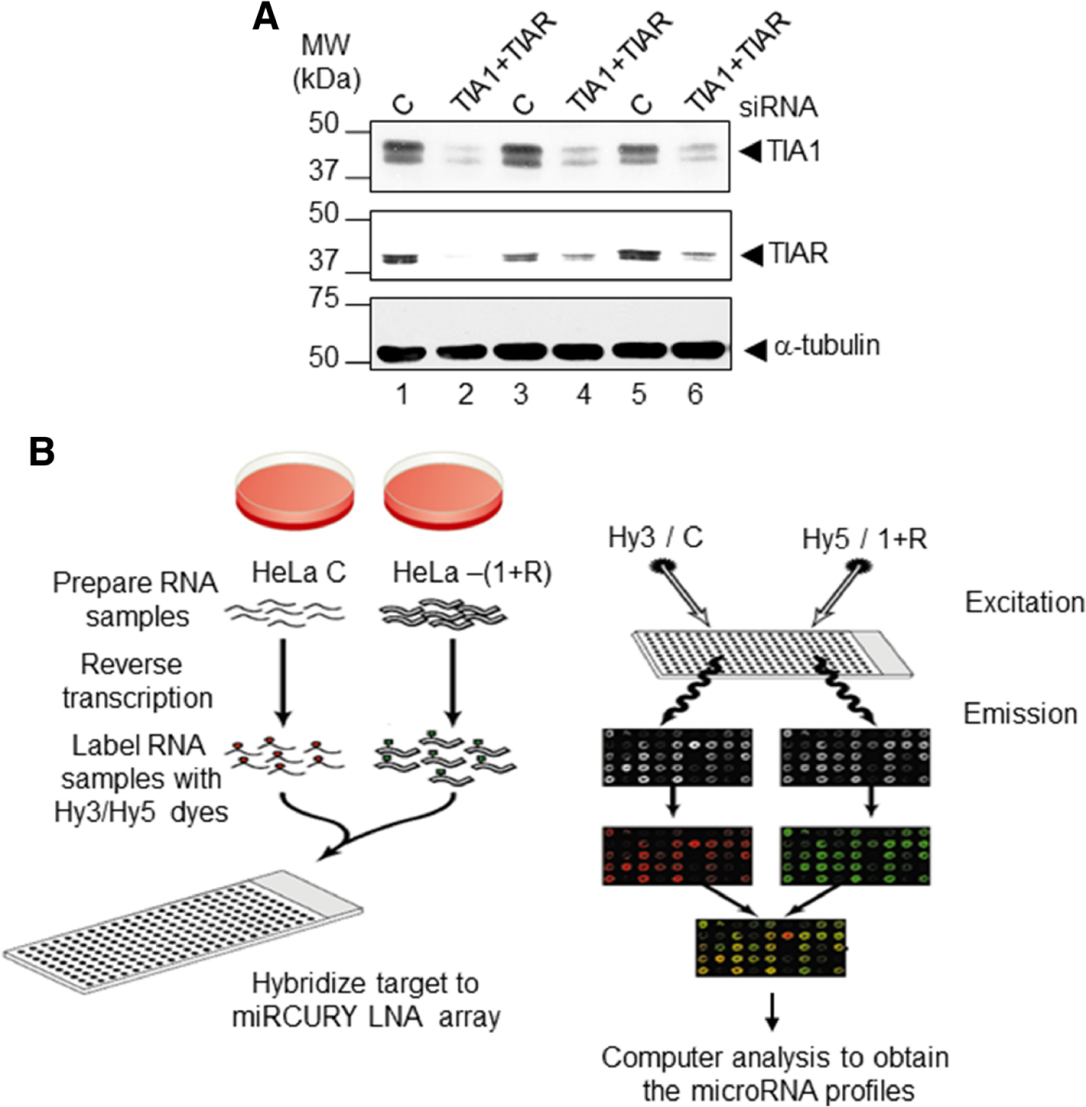
**Small interfering RNA (siRNA)-mediated depletion of TIA proteins. **(**A**) Western blot analysis of HeLa cell lysates prepared 72 h after transfection with siRNAs against control (C; lanes 1, 3 and 5) and TIA1 plus TIAR (lanes 2, 4 and 6). The blot was probed with antibodies against TIA1, TIAR, and α-tubulin proteins, as indicated. Molecular weight markers and the identities of protein bands are shown. (**B**) A schematic representation depicting the miRNA array strategy used in this study. The preparation of the RNA samples from control and TIA-depleted HeLa cells, labeling of above RNA samples with Hy3 and Hy5 dyes, hybridization and analyses of the resulting miRNA profiles are illustrated.

To analyze the putative role of TIA proteins in the control of gene expression on microRNAs, we transfected HeLa cells with double-stranded small interfering RNAs (siRNAs) targeting TIA1 and TIAR mRNAs or with control siRNA (C), as previously reported [10]. The effect of siRNAs on TIA1/TIAR expression was analyzed by Western blotting (Figure 1A). Upon TIA1/TIAR-RNA interference-based silencing, 80-90% and 70-80% depletions of TIA1 and TIAR proteins, respectively (Figure 1A) were achieved 72 h after transfection, thus in agreement with previous observations [10]. By contrast, α-tubulin protein (Figure 1A) was used as control of siRNA specificity, and its expression level was not significantly affected by gene interference approach.

To test the microRNA (miRNA) expression profiles resulting from the reduction of TIA1 and TIAR protein levels, the differences in global miRNA expression patterns in control and TIA1/TIAR-depleted HeLa cells were determined by means of a miRCURY™ LNA Array with specific probes for simultaneous analysis of at least 600 different miRNAs. After having passed sample QC on the Bioanalyzer2100 and RNA measurement on the Nanodrop instrument, the samples were labelled using the miRCURY™ Hy3™/Hy5™ labelling kit and hybridized on the miRCURY™ LNA Array (v.8.1) (Figure 1B). Analysis of the scanned slides showed that the labelling was successful as all capture probes for the control spike-in oligo nucleotides produced signals in the expected range. The quantified signals were normalized using the global Lowess (Locally WEighted Scatterplot Smoothing) regression algorithm, which we have found produces the best within-slide normalization to minimize the intensity-dependent differences between the dyes (Figure 1B). The positive effect of this normalization is illustrated in 3 different plots for each experimental conditions analyzed (Additional file 1). It is interesting to note the high reproducibility of individual miRNA expression levels and their correlation across the different miRNA pools (Additional file 1). On the other hand, an appropriate statistical test analysis was made using a linear model (as implemented in the limma R/Biocounductor package) to compare miRNA expression patterns for three two-color arrays performed from three independent biological replicates (Figure 1 and Additional file 1).

As shown in Table 1, depletion of TIA-proteins resulted in significantly altered miRNA expression profiling. The expression level of 29 out of 600 miRNAs was found to more than double when comparing channels Hy5 and Hy3. The genome-wide profiling analysis identified 17 miRNAs and 12 putative in-silico miRNAs (identified as miRPlus). These were the well-established miRNAs: miR-197-3p, miR-210, miR-373-5p, miR-492, miR-498, miR-503-5p, miR-572, miR-586, miR-612, miR-615-3p, miR-623, miR-625-5p, miR-638, miR-658, miR-663a, miR-671-5p and miR-769-3p, which were differentially up-regulated at least 2-fold (FDR<0.0001). The identified miRPlus sequences were in licensed human sequences; now a great number of these sequences have been annotated in the corresponding miRNA database. This information is available in Table 1. Regarding the differentially expressed miRPlus in our experimental conditions, an update of these miRPlus sequences in the miRBase 18 indicates that miRPlus-17836 is miR-30b-3p, miRPlus-17864 is miR-744-5p, miRPlus-17867 is miR-203a, miRPlus-17877 and miRPlus-17960 are miR-483-5p, miRPlus-17878 is miR-193a-5p, miRPlus-17942 is miR-125a-3p, miRPlus-17950 is miR-371-5p and miRPlus-17961 is miR-629-5p (Table 1). The remaining miRPlus sequences have not been yet assigned to the putative specific miRNAs (Additional file 2).

**Table 1:** MicroRNA expression profiling in TIA-depleted HeLa cells

**Fold change**	**pval (LiMMA)**	**FDR (LiMMA)**	**miRNA ID**	**miRNA ID update**
2.21	4.13E-06	8.26E-05	hsa-miR-197_MM2	hsa-miR-197-3p
3.84	5.00E-07	1.70E-05	hsa-miR-210	-
2.3	4.84E-06	9.38E-05	hsa-miR-373*	hsa-miR-373-5p
2.75	3.78E-06	7.77E-05	hsa-miR-492	-
2.79	1.38E-06	3.55E-05	hsa-miR-498	-
2.5	5.22E-06	9.89E-05	hsa-miR-503	hsa-miR-503-5p
4.29	1.00E-07	4.05E-06	hsa-miR-572	-
3.78	3.60E-07	1.27E-05	hsa-miR-586	-
2.24	5.01E-06	9.59E-05	hsa-miR-612	-
2.82	4.50E-06	8.81E-05	hsa-miR-615	hsa-miR-615-3p
3.18	1.91E-06	4.50E-05	hsa-miR-623	-
2.84	1.23E-06	3.31E-05	hsa-miR-625	hsa-miR-625-5p
2.11	3.63E-06	7.54E-05	hsa-miR-638	-
2.44	1.64E-06	3.96E-05	hsa-miR-658	-
3.61	2.57E-06	5.63E-05	hsa-miR-663	hsa-miR-663a
2.23	2.75E-06	5.96E-05	hsa-miR-671	hsa-miR-671-5p
3.65	3.18E-06	6.70E-05	hsa-miR-769-3p	-
3.44	2.60E-07	9.88E-06	miRPlus_17832	n.d.
2.73	9.60E-07	2.77E-05	miRPlus_17836	hsa-miR-30b-3p
4.38	5.70E-07	1.91E-05	miRPlus_17856	n.d.
3.34	2.26E-06	5.13E-05	miRPlus_17864	hsa-miR-744-5p
2.5	2.91E-06	6.23E-05	miRPlus_17867	hsa-miR-203a
2.7	6.30E-07	2.04E-05	miRPlus_17877/17960	hsa-miR-483-5p
2.33	4.69E-06	9.13E-05	miRPlus_17878	hsa-miR-193a-5p
2.84	1.87E-06	4.43E-05	miRPlus_17881	n.d.
3.63	5.50E-07	1.85E-05	miRPlus_17890	n.d.
3.3	2.16E-06	4.94E-05	miRPlus_17942	hsa-miR-125a-3p
2.15	2.85E-06	6.14E-05	miRPlus_17950	hsa-miR-371-5p
2.6	1.34E-06	3.45E-05	miRPlus_17961	hsa-miR-629-5p

miRNA cluster defining a signature of up-regulated miRNAs in TIA-depleted HeLa cells. The microarray data were analyzed by the limma R method. Fold is an average measure of the fold change in differential expression and the false discovery rate (FDR) indicates the expected percentage of false positives (FDR < 0.0001). miRNA ID update are miRNAs and miRPlus renamed in agreement with miRBase 18 database. n.d. means non-determined.

**Figure 2:**
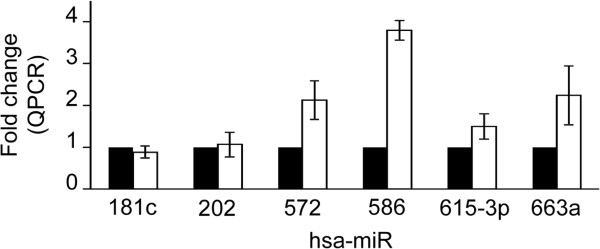
**Validation of miRNA array-predicted changes by quantitative RT-PCR (QPCR). **Quantitative miRNA expression analysis was carried out using TaqMan probes by QPCR. The represented values were normalized and expressed relative to control values (whose value was fixed arbitrarily to 1) as ratios and are means ± SD (n = 2).

To validate previous results on identified miRNAs and their relative expression levels, four up-regulated and two unchanged miRNAs were confirmed by TaqMan reverse transcription and polymerase chain reaction (QPCR) analyses. As shown in Figure 2, the results obtained by quantitative amplification were fully comparable and the relative fold changes in miRNA expression were consistent with the data detected by hybridization in the corresponding microarrays (compare Table 1 and Figure 2).

**Figure 3:**
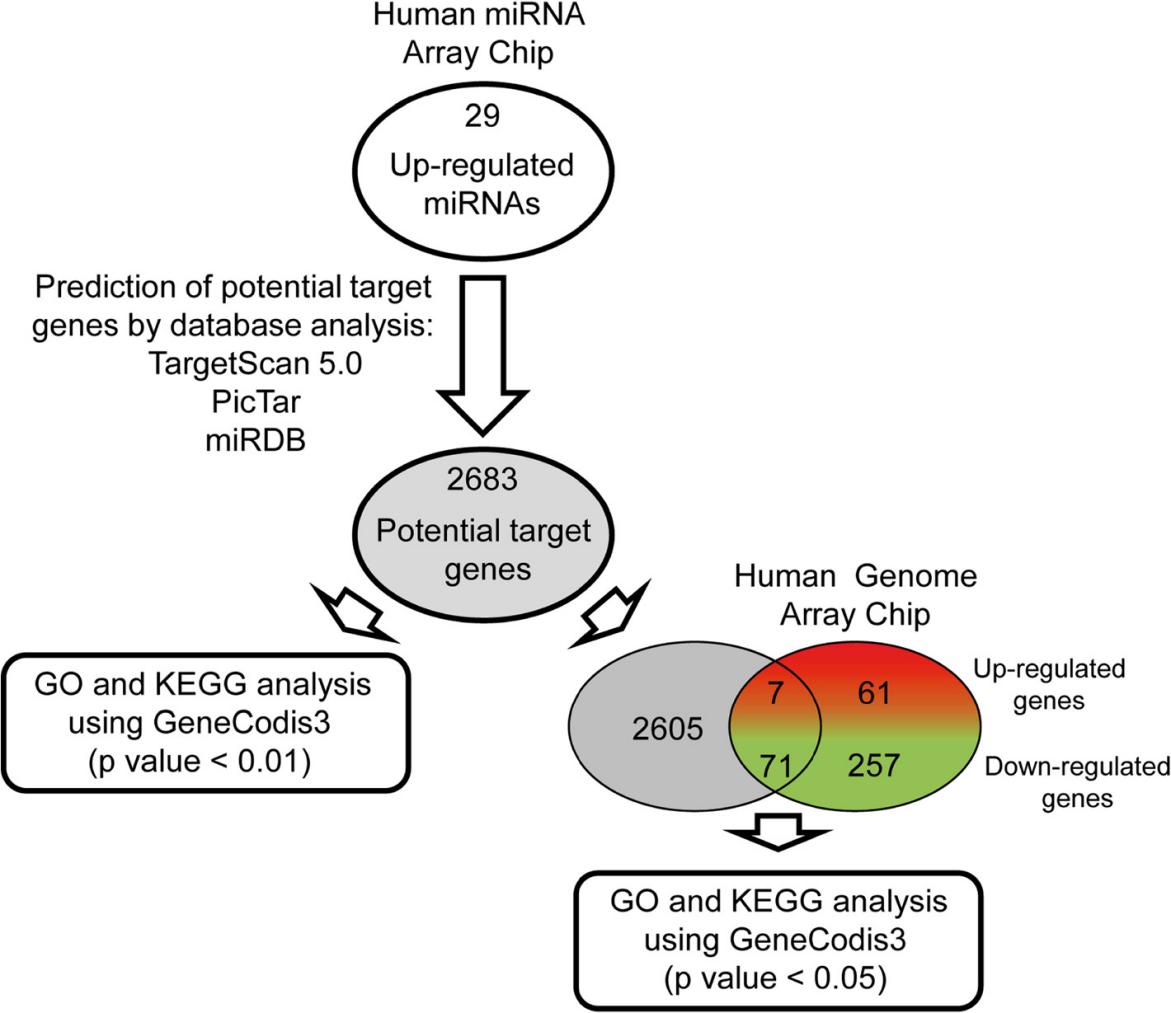
**Prediction of potential and experimental target genes associated with up-regulated miRNAs in TIA-depleted HeLa cells. **Integrative analyses of potential target genes and miRNAs regulated in TIA-depleted HeLa cells using TargetScan 5.2, PicTar-Vert and miRDB software tools (see corresponding section in Methods). Diagrams depicting the number of potential target genes and up-regulated miRNAs (Additional file 3) by TIA silencing are shown. Venn diagram depicting the numbers of genes that were intersected among putative target genes (highlighted in gray) associated to miRNAs and experimentally defined differentially expressed genes (see additional data files in [10]) in HeLa cells lacking TIA proteins. The Gene Ontology (GO) and Kyoto Encyclopedia of Genes and Genomes (KEGG) database analyses were conducted using software programmes provided by GenCodis3.

As a first attempt to understand the relevance of 29 differentially up-regulated miRNAs, in silico methods for predicting human miRNA target genes were used (Figure 3). The potential target genes were identified by searching the TargetScan, PicTar and miRBD databases [25-28]. By comparing and selecting alone non-repeated target genes, 2683 miRNA target genes were identified (Additional file 3). All these potential target genes were tested using computer tool GeneCodis3 [20,21] to elucidate the biological processes and cellular pathways assigned using Gene Ontology (GO) and Kyoto Encyclopedia of Genes and Genomes (KEGG) database analyses (Figure 3 and Additional file 3). A total of 253 biological processes from GO database and 50 cellular pathways from KEGG categories were estimated with a significance hypergeometric test (corrected hypergeometric p value < 0.01). Target genes corresponding to the biological processes from GO categories were mainly involved in regulation of DNA-dependent transcription, signal transduction, multicellular organismal development, positive/negative regulation of transcription from RNA polymerase II promoter, cell adhesion, transmembrane transport, apoptotic process and nervous system development (Table 2 and Additional file 3). In addition, target genes associated to the main biological pathways were also identified using KEGG database involving pathways in cancer, MAPK signalling pathway, focal adhesion, endocytosis, regulation of actin cytoskeleton, Wnt signalling pathway, neuroactive ligand-receptor interaction, glutamatergic synapse, ubiquitin mediated proteolysis and tuberculosis (Table 2 and Additional file 3). Collectively, these results suggest that TIA-protein depletion promotes the induction of a miRNA signature which may directly and indirectly contribute to the establishment of the observed cellular phenotypes in TIA-deficient HeLa cells [10].

**Table 2:** Top ten biological processes and pathways associated to up-regulated miRNAs in TIA-depleted HeLa cells

**Term**	**Description**	**Number of genes**	**p-value**
**GO**			
GO:0006355	Regulation of transcription, DNA-dependent	269	2.49E-33
GO:0007165	Signal transduction	188	1.00E-18
GO:0007275	Multicellular organismal development	151	5.17E14
GO:0045944	Positive regulation of transcription from RNA pol II promoter	124	2.69E-21
GO:0007155	Cell adhesion	103	4.06E-12
GO:0055085	Transmembrane transport	102	5.89E-08
GO:0045893	Positive regulation of transcription, DNA-dependent	96	5.89E-14
GO:0006915	Apoptotic process	96	4.13E-07
GO:0000122	Negative regulation of transcription from RNA pol II promoter	95	4.66E-17
GO:0007399	Nervous system development	93	1.98E-16
**KEGG**			
Kegg:05200	Pathways in cancer	68	5.72E-11
Kegg:04010	MAPK signaling pathway	58	3.68E-08
Kegg:04510	Focal adhesion	49	2.75E-08
Kegg:04144	Endocytosis	46	7.60E-07
Kegg:04810	Regulation of actin cytoskeleton	44	1.48E-04
Kegg:04310	Wnt signaling pathway	37	3.88E-05
Kegg:04080	Neuroactive ligand-receptor interaction	35	9.43E-04
Kegg:04724	Glutamatergic synapse	32	2.34E04
Kegg:04120	Ubiquitin mediated proteolysis	31	5.96E-03
Kegg:05152	Tuberculosis	28	1.17E-04

Gene Ontology (GO) and Kyoto Encyclopedia of Genes and Genomes (KEGG) databases analysis were carried out using GeneCodis3 software. The categories were ranked on their numbers of associated genes and the ten with the highest number of genes are shown.

On the other hand, the collection of differentially expressed genes previously identified by expression microarray Human Genome U133 Plus 2.0 (Affymetrix) in TIA-depleted HeLa cells [10] were intersected with potential target genes identified using in silico target prediction tools. The results show that only 7 out of 68 (10%) up- and 71 out of 328 (22%) down-regulated genes were shared (Figure 3 and Table 3). GO and KEGG database analyses were independently performed for up- and down-regulated genes with GeneCodis3 software. The results suggest that the enrichment functional categories (p < 0.05) are related with signal transduction, innate immune response and response to interleukin-1 for up-regulated genes and glomerular basement membrane development for down-regulated genes. Further, cellular pathways of neuroactive lingand-receptor interaction and apoptosis were associated to up-regulated genes, whereas endocytosis and lysosomes were linked to down-regulated genes (Table 4). Taken together, these results suggest that at least a fraction (40%) of the up-regulated miRNAs (12 out of 30) could be contributing to the establishment of differential expression profiles associated to the HeLa cells lacking TIA proteins.

**Table 3:** Intersection between potential and experimentally defined target genes related to miRNAs in TIA-depleted HeLa cells

**Gene symbol**	**Description**	**Associated miRNA**	**TIA-iCLIP**
**Up-regulated genes**			
CNR1	Cannabinoid receptor 1 (brain)	miR-30b-3p	+
EIF4A2	Eukaryotic translation initiation factor 4A, isoform 2	miR-586	+++
EREG	Epiregulin	miR-586	+
F2RL2	Coagulation factor II (thrombin) receptor-like 2	miR-30b-3p	+
IL1R1	Interleukin 1 receptor, type I	miR-498	++
IRAK2	Interleukin-1 receptor-associated kinase 2	miR-503-5p	++
SELI	Selenoprotein I	miR-197-3p	++
**Down-regulated genes**			
ACADSB	Acyl-Coenzyme A dehydrogenase, short/branched chain	miR-203	++
ACOX1	Acyl-Coenzyme A oxidase 1, palmitoyl	miR-373-5p	++
ALS2CR4	Amyotrophic lateral sclerosis 2 (juvenile) chromosome region, candidate 4	miR-203	+++
ANKH	Ankylosis, progressive homolog (mouse)	miR-203	+++
AP1S2	Adaptor-related protein complex 1, sigma 2 subunit	miR-203	++
AP2B1	Adaptor-related protein complex 2, beta 1 subunit	miR-203	+++
APPBP2	Amyloid beta precursor protein (cytoplasmic tail) binding protein 2	miR-612	++
BBS1	Bardet-Biedl syndrome 1	miR-612	+
BRIP1	BRCA1 interacting protein C-terminal helicase 1	miR-373-5p	+
C16orf72	Chromosome 16 open reading frame 72	miR-671-5p	+++
C18orf54	Chromosome 18 open reading frame 54	miR-625-5p	++
C1orf96	Chromosome 1 open reading frame 96	miR-373-5p	++
C20orf108	Chromosome 20 open reading frame 108	miR-30b-3p	+
CCDC50	Coiled-coil domain containing 50	miR-203	+
CENPH	Centromere protein H	miR-612	+
CLCC1	Chloride channel CLIC-like 1	miR-373-5p and 30b-3p	++
COL4A4	Collagen, type IV, alpha 4	miR-203	+++
CTDSPL2	CTD (carboxy-terminal domain, RNA polymerase II, polypeptide A) small phosphatase like 2	miR-203	+++
CTSC	Cathepsin C	miR-203	++
DAB2	Disabled homolog 2, mitogen-responsive phosphoprotein (Drosophila)	miR-203	+++
DDIT4	DNA-damage-inducible transcript 4	miR-30b-3p	++
EEF1A1	Eukaryotic translation elongation factor 1 alpha 1	miR-373-5p	+
EIF4EBP2	Eukaryotic translation initiation factor 4E binding protein 2	miR-373-5p	++
ELMOD2	ELMO/CED-12 domain containing 2	miR-30b-3p	++
EPHA7	EPH receptor A7	miR-503-5p	+++
FAM129A	Family with sequence similarity 129, member A	miR-373-5p and 586	++
FBXO9	F-box protein 9	miR-203	++
IQCE	IQ motif containing E	miT-483-5p	+
KCTD12	Potassium channel tetramerisation domain containing 12	miR-373-5p and 586	+
KLF9	Kruppel-like factor 9	miR-373-5p	++
KRIT1	KRIT1, ankyrin repeat containing	miR-373-5p	+
LAMP2	Lysosomal-associated membrane protein 2	miR-373-5p	++
MCM4	Minichromosome maintenance complex component 4	miR-373-5p	++
MECP2	Methyl CpG binding protein 2 (Rett syndrome)	miR-203	+
MIB1	Mindbomb homolog 1 (Drosophila)	miR-373-5p, 503 and 203	++
MOBKL2B	MOB1, Mps One Binder kinase activator-like 2B (yeast)	miR-503-5p and 203	+++
MTAP	Methylthioadenosine phosphorylase	miR-125a-3p	++
MTHFD2L	Methylenetetrahydrofolate dehydrogenase (NADP+ dependent) 2-like	miR-373-5p	++
NAV1	Neuron navigator 1	miR-503-5p	++
NDRG3	NDRG family member 3	miR-203	++
NDST1	N-deacetylase/N-sulfotransferase (heparan glucosaminyl) 1	miR-30b-3p	+
NHLRC2	NHL repeat containing 2	miR-373-5p	++
NID1	Nidogen 1	miR-30b-3p	++
PAWR	PRKC, apoptosis, WT1, regulator	miR-30b-3p	+++
PCGF6	Polycomb group ring finger 6	miR-203	+
PDE1A	Phosphodiesterase 1A, calmodulin-dependent	miR-373-5p	+++
PGM2	Phosphoglucomutase 2	miR-498	+++
PLD1	Phospholipase D1, phosphatidylcholine-specific	miR-203	+++
RAB22A	RAB22A, member RAS oncogene family	miR-498	++
RBM8A	RNA binding motif protein 8A	miR-373-5p	+
RECK	Reversion-inducing-cysteine-rich protein with kazal motifs	miR-503-5p	++
RGC32	Regulator of cell cycle	miR-30b-5p	++
SFPQ	Splicing factor proline/glutamine-rich (polypyrimidine tract binding protein associated)	miR-586	++
SGPL1	Sphingosine-1-phosphate lyase 1	miR-373-5p	++
SLC12A2	Solute carrier family 12 (sodium/potassium/chloride transporters), member 2	miR-503-5p, 586 and 203	+
SLC35B3	Solute carrier family 35, member B3	miR-203	++
SNAPC3	Small nuclear RNA activating complex, polypeptide 3, 50kDa	miR-373-5p and 671	+++
STXBP4	Syntaxin binding protein 4	miR-625-5p	+
SUDS3	Suppressor of defective silencing 3 homolog (S. cerevisiae)	miR-203	+++
SYNC1	Syncoilin, intermediate filament 1	miR-203	++
TFDP2	Transcription factor Dp-2 (E2F dimerization partner 2)	miR-30b-3p and 203	+++
TIA1	TIA1 cytotoxic granule-associated RNA binding protein	miR-30b3p	+++
TNFRSF19	Tumor necrosis factor receptor superfamily, member 19	miR-125a-3p	++
TNRC6B	Trinucleotide repeat containing 6B	miR-503-5p, 586 and 203	++
TSEN2	tRNA splicing endonuclease 2 homolog (S. cerevisiae)	miR-197-3p	+
VAMP1	Vesicle-associated membrane protein 1 (synaptobrevin 1)	miR-203	++
VGLL3	Vestigial like 3 (Drosophila)	miR-373-5p	++
VPS13A	Vacuolar protein sorting 13 homolog A (S. cerevisiae)	miR-586	++
WDFY3	WD repeat and FYVE domain containing 3	miR-203	+
ZBTB44	Zinc finger and BTB domain containing 44	miR-203	++
ZNF169	Zinc finger protein 169	miR-125a-3p	++

Gene cluster defining a molecular signature of up- (highlighted in red in Figure 3) and down-regulated (highlighted in green in Figure 3) genes linked to up-regulated miRNAs in TIA-depleted HeLa cells. Gene symbol, gene title as description and associated miRNAs are indicated. Estimation of the density of binding sites of TIA proteins on up- and down-regulated target genes by iCLIP analysis is shown. The relative quantification estimated as low (+), medium (++) and high (+++) density is indicated.

**Table 4:** Biological processes and pathways linked to experimentally defined and differentially expressed genes in TIA-depleted HeLa cells

**Categories enriched in**	**Description**	**Number of genes**	**Gene symbol**	**p-value**
**Up-regulated genes**				
**GO term**				
GO:0070555	Response to interleukin-1	2	IL1R1, IRAK2	6.21E-04
GO:0045087	Innate immune response	2	IL1R1, IRAK2	6,88E-03
GO:0007165	Signal transduction	2	IL1R1, IRAK2	2,88E-02
**KEGG term**				
Kegg:04080	Neuroactive ligand-receptor interaction	2	CNR1, F2RL2	6.88E-03
Kegg:04210	Apoptosis	2	IL1R1, IRAK2	1.43E-03
**Down-regulated genes**				
**GO term**				
GO:0032836	Glomerular basement membrane development	2	COL4A4, NID1	1.09E-02
**KEGG term**				
Kegg:04144	Endocytosis	4	RAB22A, AP2B1, DAB2, PLD1	3.23E-02
Kegg:04142	Lysosome	3	CTSC, AP1S2, LAMP2	4.77E-02

Gene Ontology (GO) and Kyoto Encyclopedia of Genes and Genomes (KEGG) databases analysis were carried out using GeneCodis3 software.

**Figure 4:**
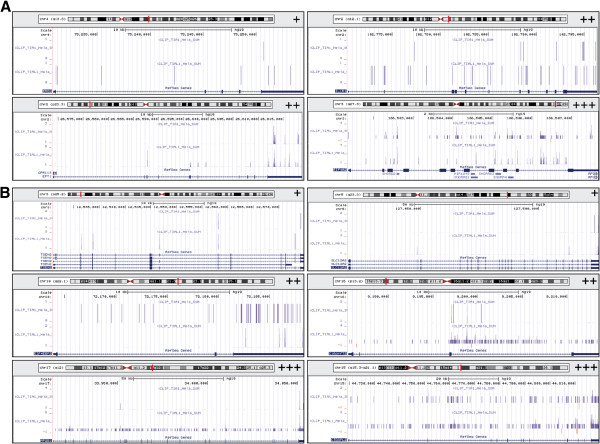
**RNA map of TIA proteins in targeted pre-mRNAs related with miRNAs in TIA-deficient HeLa cells. **(**A** and **B**) Profiles of experimental crosslink sites of TIA1 and TIAR proteins (adapted from TIA-iCLIP analysis [8]) on up- (**A**) and down-regulated (**B**) pre-mRNAs in TIA-depleted HeLa cells. Representative examples of up- (**A**) and down-regulated (**B**) genes with low (+), medium (++) and high (+++) density of TIA binding sites are shown. The bar graph in each panel indicates the number of cDNAs that identified each crosslink site.

Based on above observations an interesting question emerges: why should many specific genes be up- or down-regulated by induced miRNAs in TIA-depleted HeLa cells? A simple answer to this question might be that these mRNAs or their precursors (pre-mRNAs) are targeted by TIA proteins through one or multiple layers to exert the control of their gene expression; thus, these regulators can act as multifunctional RNA binding proteins (see references included in Background). To approach this issue, the experimental profiles of the binding patterns of TIA proteins (i.e., the RNA map corresponding to TIA proteins) at the up- or down-regulated pre-mRNAs (Figure 4 and Table 3) were examined using the iCLIP database of TIA proteins kindly provided by Jernej Ule’s laboratory [8]. For example, as shown in Figure 4 and in Table 3, the pre-mRNAs analyzed show multiple sequence sites located across the full-length pre-mRNAs, both exons and introns, and which we have classified as genes with either low (+), medium (++) or high (+++) density of TIA binding sites. The greatest relevance of this observation is the fact that the density of binding sites on these pre-mRNAs is found in both up- and down-regulated genes and located with frequency on the last exons of these pre-mRNAs and particularly on the sequences located at the 3^′^ untranslated regions of the mature mRNAs. In this regard, it is reasonable to think that the existence of a feedback loop that represses the expression of many genes, which could be activated in the absence of TIA proteins, for example at the post-transcriptional levels (i.e. mRNA stability or translational activation), to dampen its expression in order to promote or counteract the cellular phenotypes associated to the absence of the TIA proteins.

**Figure 5:**
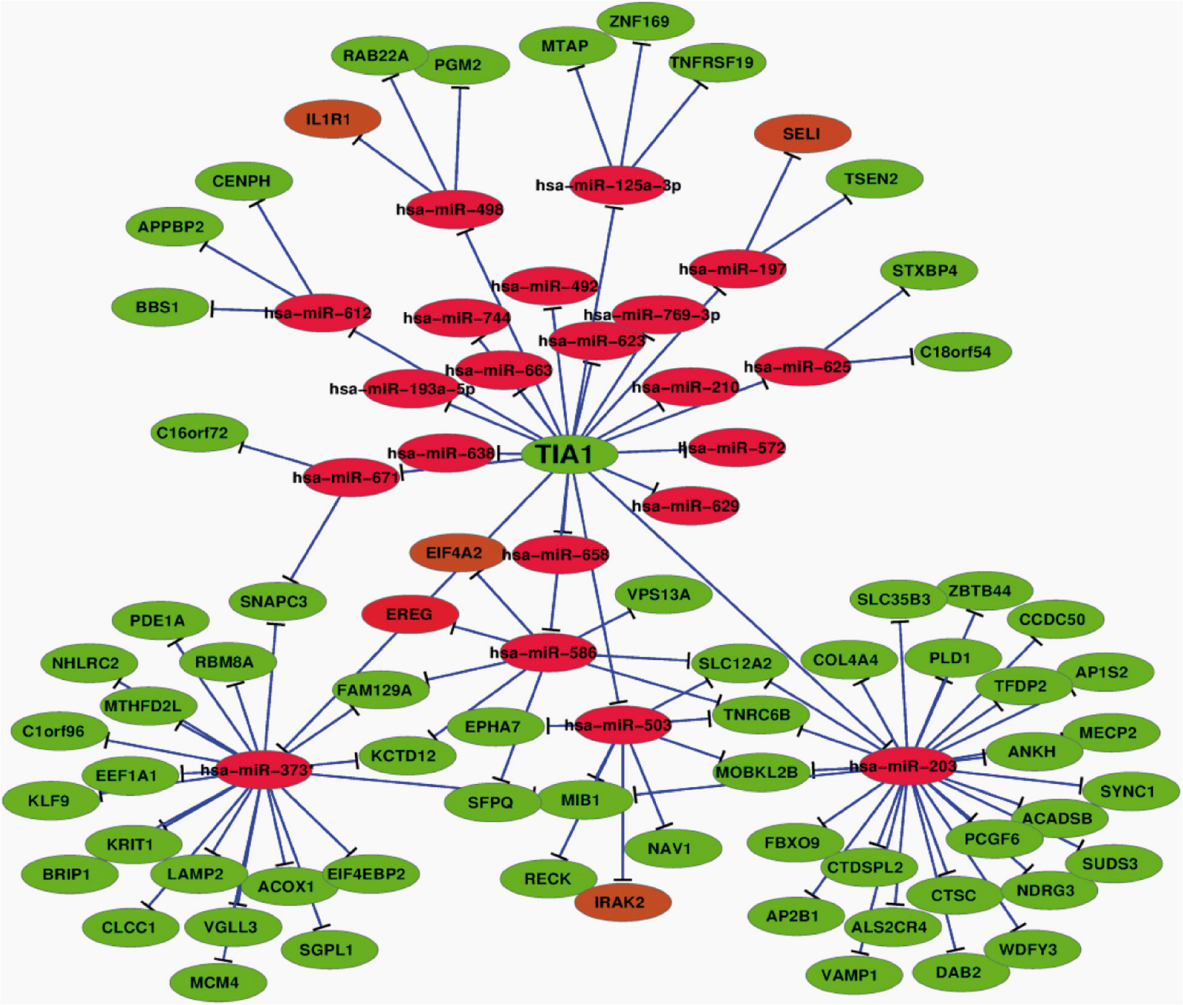
**Regulatory network of up-regulated miRNAs and intersection with target genes associated with TIA-depleted HeLa cells. **Elongated circles represent differentially expressed miRNAs and genes (red and brown- increased, green- decreased). Lines represent regulatory relations between differentially expressed miRNAs and genes.

During the last decade, much progress has been made in the understanding of network topology and the relevance and properties of its basic modules. In this study, we analyze and assess module networks inferred from both miRNAs and gene expression data using a bioinformatic tool as CytoScape [32]. By matching expressed miRNAs and experimentally defined up- and down-regulated genes in TIA-depleted HeLa cells, a putative regulatory network of TIA-associated genes and miRNAs was constructed. Based on the number of potential gene interactions with single miRNAs, both up- and down-expressed genes regulating TIA-mediated differential expression were connected (Figure 5). This network draws a cellular scenario where the reduction of TIA proteins could lead to molecular responses mediated by individual miRNAs. Such a computational approach, starting from expression data alone, can be helpful in the future process of identification of the function of these miRNAs by suggesting modules of co-expressed genes in which they could play a regulatory role.

## Discussion

Current data suggest that a significant portion of the information containing the human genome is regulated by miRNAs. These small RNAs recognize and regulate target genes. In this regard, more than 60% of protein-coding genes are predicted *in silico* as targets, based on conserved base-pairing between the 3^′^- and 5^′^-untranslated regions (UTR) of the mRNA and the seed sequence of the miRNAs [33] without considering putative target on coding sequences. At present, near to 2,000 miRNAs have been identified in the human genome and about 20-30% of human genes are controlled by one or more miRNAs [33-36]. Multiple lines of evidence indicate that they are key regulators of numerous critical functions in development and disease, including cancer. Many of them have been reported to have molecular features and act either as tumour suppressors or oncogenes [33-35]. The changes in miRNA signature identified in this study might directly and indirectly function as encouraging/counteracting mechanism of biological processes and cellular pathways to promote/attenuate the inflammatory, angiogenic and proliferative responses linked to TIA-depleted HeLa cells [10].

**Figure 6:**
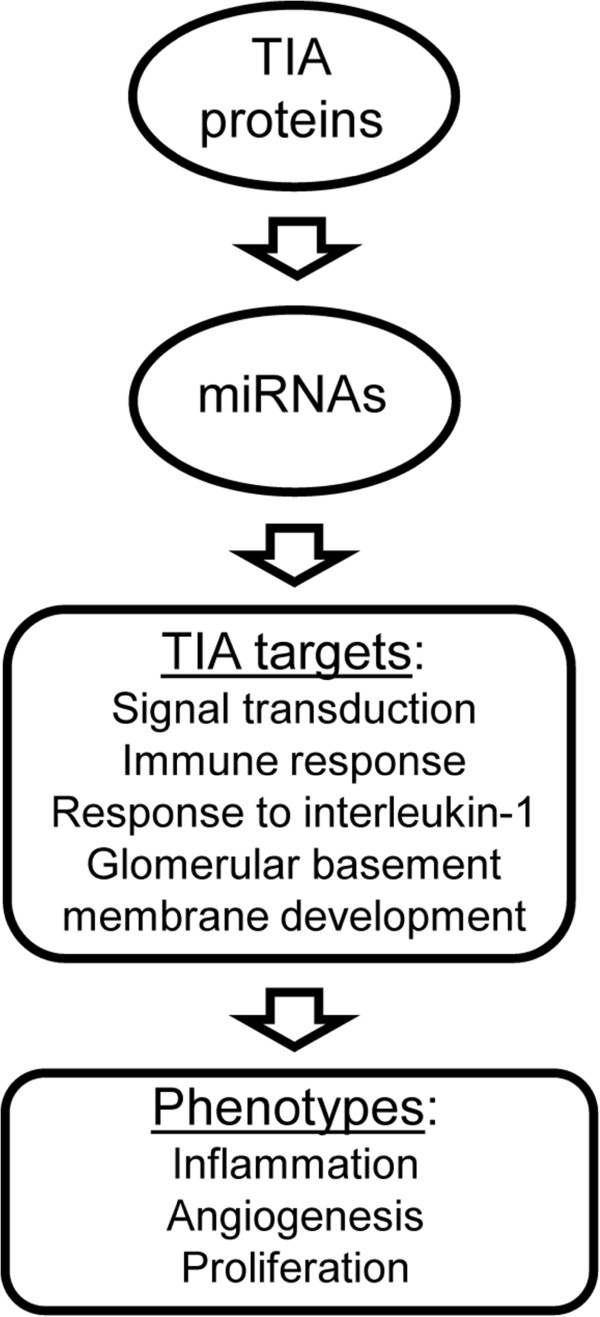
**An overview summarizing the cellular and molecular events associated to the reduction of TIA proteins. **The availability of TIA proteins could be contributing to a regulatory loop mediated by miRNAs to modulate target gene expression related to inflammatory, angiogenic and/or proliferative responses/phenotypes in HeLa cells.

Given that many target genes associated with the identified up-regulated miRNAs are down-regulated (Table 3) in TIA-depleted HeLa cells [10], from a mechanistic viewpoint our results indicate that mRNA abundance in most targeted genes was somewhat affected by miRNAs, thus suggesting that, for a substantial number of genes regulated by TIA-protein absence, destabilization of mRNA may be the main mechanism of protein repression by these miRNA-mediated regulators. This observation agrees with a recent study, suggesting that mammalian miRNAs predominantly reduce target mRNA levels [34]. However, some miRNAs such as miR-744 enhances cyclin B1 mRNA expression through a novel mechanism. miRNA positively-regulates gene expression by targeting promoter elements; this phenomenon is known as RNA activation [37]. In this regard, it is reasonable to think that this mechanism could be operating in the up-regulation observed for seven target genes associated with the up-regulated miRNAs (Table 3). Furthermore, miRNAs do not only regulate the expression of protein-encoding genes but also other miRNAs: for instance, let-7a controls the expression of important epigenetic regulators, including epigenetic miRNA regulatory circuits, and organizes the whole gene expression profile [38]. On the other hand, there are miRNA-target interactions that involve multiple sites for a given target and confer much stronger repression. More often, different miRNAs work together to co-target a given mRNA, therefore their combined repressive effect greatly exceeds the individual contributions [34-36]. Both regulatory situations are observed in the down-regulated genes associated to up-regulated miRNAs in TIA-depleted HeLa cells (Table 3). This suggests that clusters of miRNAs can play a more prominent role than only reinforcing the expression patterns dictated by transcriptome dynamics. The existence of interactions among these regulators and the interactions between their regulatees suggests that these miRNAs generate networks that modulate antagonistic cellular responses, such as apoptosis or cell proliferation induction and/or repression. Our set of data indicates that the action of miRNAs could potentially be another important mechanism in the regulation of gene expression and some gene regulatory networks mediated by TIA proteins. These observations thus suggest the existence of feedback mechanisms that promote miRNA expression, which might therefore contribute to dampen the phenotypes observed in TIA-lacking HeLa cells. A type 2 incoherent feedforward loop [39] may contribute to the repression model between TIA proteins, target genes and associated miRNAs. This feedforward loop could reinforce the functional role of TIA proteins as repressors of inflammatory, angiogenic, and proliferative responses (Figure 6).

Regarding some miRNAs identified in this study, these could be related to miRNA-mediated gene expression fluctuations and, more faithfully, to signal outcomes in the context of specific regulatory networks. This suggest that miRNAs can strenghten transcriptome patterns by buffering the deleterious effects of some network states linked to random fluctuations in gene expression program, in agreement with previous findings [25]. Since little experimental information is available on potential target genes and miRNA cluster identified in our study and we have not experimentally carried out analysis of gain- and loss-of function, we used experimental data on miRNA function to understand the potential implication and relevance of these miRNAs in the development and progression of (patho)-physiological conditions. For example, miRNA-30b has been implicated in angiogenesis [40], TRAIL-induced apoptosis in glioma cells [41] and oral squamous cell cancers [42]. miR-193a-5p targets YY1-APC regulatory axis in human endometrioid endometrial adenocarcinoma [43]. miR-203 participates in a regulatory network that modulates epithelial to mesenchymal transition [44] as well as to promote suppression immune [45]. miR-210 is induced under hypoxia and works as a iron sensor to stimulate cell proliferation and promote cell survival in the hypoxic region within tumor [46-48]. Further, this miRNA modulates the mitochondrial functioning and metabolism [49,50], represses FGFRL1 and E2F3 expression, which inhibits cell apoptosis in hypoxia response [51,52], predicts poor survival in patients with breast cancer [53] and activates notch signalling pathway in angiogenesis [54]. In addition, miR-371-5p is increased in gastric cancer [55] and miR-373 functions as an oncogene in hepatocellular carcinoma given that is a new regulator of protein phosphatase 6 [56], an repressor of the large tumor suppressor homolog 2 [57] and promotes tumor invasion and metastasis in testis cancer [58,59]. Further, both miRNA-210 and miRNA-373 participate in the gene expression control of DNA repair in hypoxic stress [60]. miRNA-492 and miRNA-498 are highly expressed and regulate metastatic functions in hepatoblastoma, rectal cancer, adenocarcinoma and retinoblastoma [61-64]. In addition, miR-503 is induced in angiogenesis, down-regulates CUGBP1 and modulates metastatic function in hepatocellular cancer cell [65-68]. miRNA-572 has been involved in chronic lymphocytic leukaemia via targeting anti-apoptotic genes [69,70]. miRNA-586 is up-regulated in colorectal cancer [71]. miRNA-615-3p dysregulates CDKN2A, NF2 and JUN in malignant mesothelioma [72] and enhances the phagocytic capacity of splenic macrophages [73]. miRNA-625 promotes invasion and metastasis of gastric cancer by targeting ILK [74]. miR-629 is associated to lung cancer by targeting NBS1 [75]. miRNA-638 and miR-658 are up-regulated in cell transformation and human gastric cancer [76,77]. In addition, other miRNAs show a great potential as activators of cell proliferation/transformation phenotypes; for example miRNA-663 functions as an oncogene promoting tumorigenesis by targeting p21(WAF1/CIP1), VEGF, JunB, JunD and TGFB1 genes [78-80] and plays also a role in inflammatory response of endothelial cells [81]. miRNA 671 regulates CD44 inducing metastasis by regulating extracellular matrix functions [82] as well as miRNA-dependent gene silencing involving Ago2-mediated cleavage of a circular antisense RNA [83]. miRNA-744 up-regulates Cyclin B1 expression [37] and down-regulates Transforming Growth Factor Beta-1 expression [84]. On the other hand, other miRNAs identified in our study have bipolar features and putatively antagonistic behaviours as opposite regulators acting either positively or negatively under specific biological programmes. In this regards, there are at least 2 miRNAs showing functions to repress proliferation, angiogenesis and transformation phenotypes. This is the case of miRNA-483-5p, which suppresses the proliferation of glioma cells via directly targeting ERK1 [85] and angiogenesis in vitro by targeting serum response factor [86]. However, microRNA-483-5p and miRNA-195 have been identified as predictors of poor prognosis in adrenocortical cancer [87]. miR-125a-3p is down-regulated in non-small cell lung cancer, having inverse effects on invasion and migration of lung cancer cells [88]. This miRNA is a potent prognostic marker in gastric cancer [89]. Collectively, the miRNA expression profiling identified in this study has important aftermath in tumorigenesis and could contribute/repress to the development of cell proliferation, angiogenesis, transformation phenotypes, because individual miRNAs are associated with diagnosis, prognosis and treatment efficacy linked to the biology of human tumors. Therefore, these miRNAs can target to oncogenes or tumor suppressor genes (also they can even function themselves as such) and take part in the promotion or inhibition of tumorigenesis and cancer progression, thus being able to confer robustness to these pathological phenotypes.

In summary, our findings suggest that expression changes in individual miRNAs in TIA-depleted HeLa cells could directly or indirectly impact on biological processes and signaling pathways to favour cell phenotypes associated to the down-regulation of TIA proteins (Figure 6). Further, our observations suggest that some of the identified miRNAs are consistent with an adaptive response aimed at attenuating the inflammatory, angiogenic, and proliferative responses developed in TIA-protein absence. Therefore, cross talk between regulatory mechanisms that promote increased/reduced expression of miRNA-mediated genes may contribute to modify the relative expression levels of the mRNAs associated with TIA-lacking HeLa cells. Based on this integrative analysis, our results provide a prominent stand for future approaches aimed at characterizing the role of specific miRNAs in TIA-mediated gene expression regulation.

## Conclusions

Gene expression profiling approaches have improved our understanding on how the human transcriptome dynamics orchestrates global responses at molecular level to answer to changing environmental challenges. Our study identifies a human miRNA collection that displays significant changes in the transient absence of TIA proteins in HeLa cells. The most prominent changes are linked to the up-regulation of miR-30b-3p, miR125a-3p, miR-193a-5p, miR-197-3p, miR-203a, miR-210, miR-371-5p, miR-373-5p, miR-483-5p, miR-492, miR-498, miR-503-5p, miR-572, miR-586, miR-612, miR-615-3p, miR-623, miR-625-5p, miR-629-5p, miR-638, miR-658, miR-663a, miR-671-5p, miR-769-3p and miR-744-5p. The integration of the identified miRNAs with the potential target genes and previous gene expression data under the same experimental conditions revealed enrichment of biological processes and signaling pathways controlling relevant members of the pathways associated to the oncogenesis. Down-regulation of these cellular components may contribute to establish the inflammatory, angiogenic, and proliferative responses previously described in TIA protein-lacking HeLa cells. Therefore, our results are consistent with the existence of regulatory networks that generate correlated expressions, commonly involving miRNAs [33,90]. This regulatory architecture may increase the fidelity of inhibition of the downstream components by acting on them redundantly. In other words, a transient loss of TIA proteins can be partially compensated for by the adaptive presence of specific miRNAs. Collectively, these findings suggest that TIA proteins can act as multifunctional regulators to provide a novel meeting point between the mechanisms for cross-talking among concerted post-transcriptional regulatory layers that coordinate complex cellular responses.

## Abbreviations

iCLIP: In vivo ultraviolet (UV)-crosslinking and immunoprecipitation to identify the RNA crosslinking sites of TIA1 and TIAR proteins; GO: Gene ontology; KEGG: Kyoto and encyclopedia of genes and genomes; siRNA: Small interfering RNA; TIA1: T-cell intracellular antigen 1; TIAR/TIAL1: TIA1 related/like protein; UTR: Untranslated region of the eukaryotic mRNAs.

## Competing interests

The authors declare that they have no competing interests.

## Authors’ contributions

JMI conceived the research and designed all experiments. CSJ, IC and JB carried out the experiments and analyzed the data presented in this paper. CSJ, IC, JB and JMI wrote the paper. All authors read and approved the final manuscript.

## Supplementary Material

Additional file 1 **Summary of the miRNA array analyses. **The following additional data are available in this file: 1) Correlation of Hy3 and Hy5 signals for the spike-in controls between slides. 2) Hy5 vs Hy3 scatter, MA and ratio distribution plots (before and after normalization) and Hy5 vs Hy3 scatter plot for the spike-in controls, for each independent experiments. 3) Statistical test implemented in the limma R. Click here for file

Additional file 2 **Update of miRNAs sequences previously identified as miRPlus.** The miRPlus sequences are licensed human sequences (Exiqon, Denmark). Many of them are already annotated in the miRBase database version 18. Click here for file

Additional file 3 **List of predicted potential target genes associated to up-regulated miRNAs in transiently TIA-depleted HeLa cells. **Potential target genes of up-regulated miRNAs identified in TIA-deficient HeLa cells were predicted by TargetScan 5.2, PicTar-Vert and miRDB database downloaded using web-app miRBase (http://www.mirbase.org). The Gene Ontology (GO) and Kyoto Encyclopedia of Genes and Genomes (KEGG) database analyses were conducted using software programmes provided by GenCodis3 (http://genecodis.cnb.csic.es). Click here for file
